# Promise and peril: how health system reforms impacted public health in three Canadian provinces

**DOI:** 10.17269/s41997-023-00785-2

**Published:** 2023-07-06

**Authors:** Tamika Jarvis, Robert W. Smith, Harman Singh Sandhu, Muriel Mac-Seing, Meghan O’Neill, Laura Rosella, Sara Allin, Andrew D. Pinto

**Affiliations:** 1 Department of Health Research Methods, Evidence, and Impact, Faculty of Health Sciences, McMaster University, Hamilton, Ontario Canada; 2 North American Observatory On Health Systems and Policies, Dalla Lana School of Public Health, University of Toronto, Toronto, Ontario Canada; 3 Division of Social and Behavioural Health Sciences, Dalla Lana School of Public Health, University of Toronto, Toronto, Ontario Canada; 4 Institute of Health Policy, Management and Evaluation, Dalla Lana School of Public Health, University of Toronto, Toronto, Ontario Canada; 5 Centre for Global Health, Dalla Lana School of Public Health, University of Toronto, Toronto, Ontario Canada; 6 Population Health Analytics Lab, University of Toronto, Toronto, Ontario Canada; 7 Laboratory Medicine and Pathobiology, Temerty Faculty of Medicine, University of Toronto, Toronto, Ontario Canada; 8 Institute for Clinical Evaluative Sciences, Toronto, Ontario Canada; 9 Institute for Better Health, Trillium Health Partners, Mississauga, Ontario Canada; 10 Upstream Lab, MAP Centre for Urban Health Solutions, Li Ka Shing Knowledge Institute, Unity Health Toronto, Toronto, Ontario Canada; 11 Department of Family and Community Medicine, Faculty of Medicine, University of Toronto, Toronto, Ontario Canada; 12 Department of Family and Community Medicine, St. Michael’s Hospital, Toronto, Ontario Canada; 13 Division of Clinical Public Health, Dalla Lana School of Public Health, University of Toronto, Toronto, Ontario Canada

**Keywords:** Public health, Public health systems research, Public health administration, Organization and administration, Governance, Health workforce, Santé publique, recherche sur les systèmes de santé publique, administration de la santé publique, organisation et administration, gouvernance, ressources humaines dans le domaine de la santé

## Abstract

**Objectives:**

Several Canadian provinces and territories have reformed their health systems by centralizing power, resources, and responsibilities. Our study explored motivating factors and perceived impacts of centralization reforms on public health systems and essential operations.

**Methods:**

A multiple case study design was used to examine three Canadian provinces that have undergone, or are in the process of undergoing, health system reform. Semi-structured interviews were conducted with 58 participants within public health at strategic and operational levels, from Alberta, Ontario, and Québec. Data were analyzed using a thematic analytical approach to iteratively conceptualize and refine themes.

**Results:**

Three major themes were developed to describe the context and impacts of health system centralization reforms on public health: (1) promising “value for money” and consolidating authority; (2) impacting intersectoral and community-level collaboration; and (3) deprioritizing public health operations and contributing to workforce precarity. Centralization highlighted concerns about the prioritization of healthcare sectors. Some core public health functions were reported to operate more efficiently, with less duplication of services, and improvements in program consistency and quality, particularly in Alberta. Reforms were also reported to have diverted funding and human resources away from core essential functions, and diminished the public health workforce.

**Conclusion:**

Our study highlighted that stakeholder priorities and a limited understanding about public health systems influenced how reforms were implemented. Our findings support calls for modernized and inclusive governance, stable public health funding, and investment in the public health workforce, which may help inform future reforms.

**Supplementary Information:**

The online version contains supplementary material available at 10.17269/s41997-023-00785-2.

## Résumé

**Objectifs:**

Plusieurs provinces et territoires canadiens ont réformé leur système de santé en centralisant le pouvoir, les ressources et les responsabilités. Notre étude a exploré les facteurs sous-jacents et les impacts perçus des réformes de centralisation sur les systèmes et les opérations essentielles de santé publique.

**Méthodes:**

Nous avons mené une étude de cas multiples pour examiner la situation de trois provinces canadiennes qui ont subi ou qui sont en train de réaliser une réforme du système de santé. Des entrevues semi-structurées ont été menées auprès de 58 participants de la santé publique aux niveaux stratégique et opérationnel, en Alberta, en Ontario et au Québec. De façon itérative, nous avons thématiquement analysé les données recueillies.

**Résultats:**

Trois thèmes principaux ont été formulés pour décrire le contexte et les impacts des réformes de centralisation du système de santé sur la santé publique : 1) la promesse d’une « optimisation des ressources » et la consolidation de l’autorité, 2) l’impact sur la collaboration intersectorielle et communautaire, et 3) la privatisation des opérations de santé publique et la précarisation de la main-d’œuvre. La centralisation a mis en lumière des préoccupations quant à la priorité accordée aux services de santé. Certaines fonctions essentielles de la santé publique fonctionneraient de manière plus efficace, avec moins de dédoublement des services et des améliorations de la cohérence et de la qualité des programmes, notamment en Alberta. Les réformes auraient aussi détourné des fonds et des ressources humaines des fonctions essentielles de base et auraient réduit les effectifs de la santé publique.

**Conclusion:**

Notre étude a mis en exergue les priorités des parties prenantes et une compréhension limitée des systèmes de santé publique qui ont influencé la manière dont les réformes ont été mises en œuvre. Nos résultats soutiennent les appels à une gouvernance plus modernisée et inclusive, à un financement stable de la santé publique et à un investissement dans le personnel de santé publique, pouvant ainsi contribuer à alimenter les futures réformes.

## Introduction

In Canada, provinces and territories are responsible for administering their health systems, which include both individual-focused healthcare services and population-focused public health programs and services. Public health systems can be defined as the collection of entities in a jurisdiction, with a mandate to perform core public health operations (e.g., disease prevention, surveillance, emergency preparedness and response planning, health promotion, and health protection) and the enabling functions that support policy, programs, and services (e.g., governance, organizational structures, financing, and workforce) (Rechel et al., 2018). With the exception of public health emergencies, governments underinvest in public health compared to healthcare due in part to the impact of public health operations being less publicly visible and politically marketable (Hoffman et al., 2019). Prior to the COVID-19 pandemic, the proportion of the total health budget allocated to public health was approximately 2.8% in Alberta, 2.5% in Ontario, and 0.8% in Québec (Arpin et al., 2022; Smith et al., 2021, 2022).

**Fig. 1:**
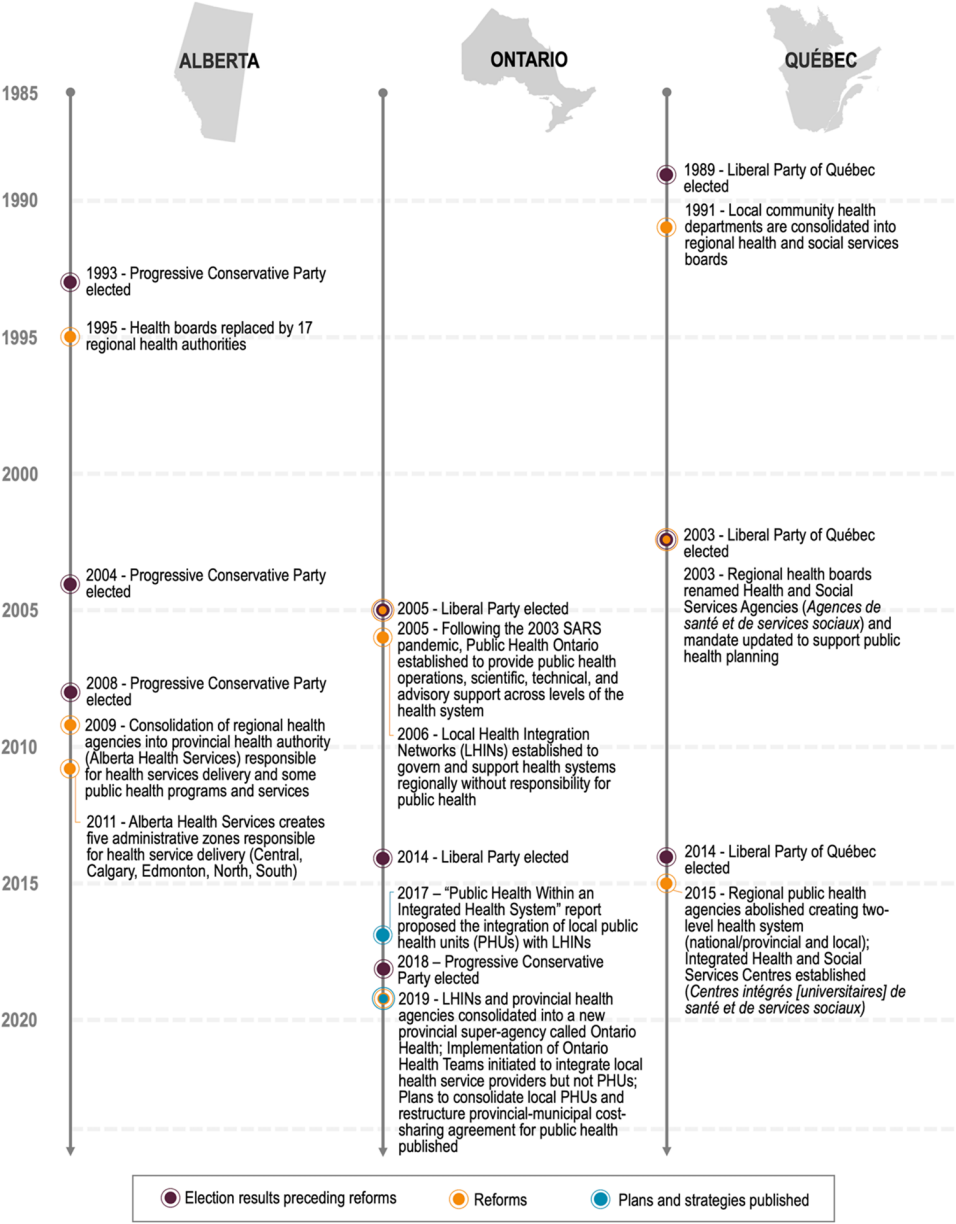
Brief timeline of recent structural reforms of provincial health systems and elections preceding them. Sources: Arpin et al., 2022; Smith et al., 2021, 2022; “List of Alberta general elections,” 2022; “List of Ontario general elections,” 2022; “List of Québec general elections,” 2022; Map outlines by Free Vector Maps [http://freevectormaps.com]

In recent years, several provinces and territories have restructured their health systems, with the stated purpose of containing costs, improving efficiency, and improving population health outcomes (Allin et al., 2018; Fierlbeck, 2019; Quesnel-Vallée & Carter, 2018). These restructuring efforts included reforms to regionalize or centralize sub-national regional health authorities, a trend that has been increasingly proposed in other jurisdictions (Duckett, 2010; Fierlbeck, 2019; Wankah et al., 2018). *Centralization* reforms shift power, resources, and responsibilities towards a higher governing authority and away from local, or more decentralized, governing bodies (Abimbola et al., 2019). The impacts of these major centralization reforms on public health systems remain unclear, and an examination of these experiences may help inform future health system reform efforts. In this paper, we examine public health leader perspectives on the context for, and impacts of, health system reforms that centralized governance and decision-making authority in three of Canada’s most populated provinces: Alberta, Ontario, and Québec. Each province examined also had different experiences with health system reforms but with trends towards centralization in all three. Figure 1 provides a summary of key structural reforms in each province.

Alberta was one of the first provinces to re-centralize its healthcare system and integrate public health functions, after successive regionalization reforms starting in the early 1990s and that continued in 2003 when 17 regional health authorities were reduced to nine (Smith et al., 2022). In 2008, just before the H1N1 influenza pandemic, Alberta Health Services (AHS) was established through the *Regional Health Authorities Act*. This act resulted in the consolidation of nine regional health agencies into AHS, a single health authority responsible for the administration and delivery of healthcare services province-wide (Smith et al., 2022). Specifically, AHS is a centralized arm’s-length delegated health authority responsible for the operations of the healthcare system and many public health programs and services.

Québec has undergone a series of health system restructuring reforms, which in most recent years, has moved towards more centralized governance authority and integrated public health into the healthcare system (Breton et al., 2009). In 2015, Québec introduced *Bill 10*, referred to as the “Barrette Reform” after Health Minister Gaétan Barrette, which amended the governance and organization of Québec’s health and social services system. This reform resulted in an estimated 30% reduction in funding to the public health sector (Arpin et al., 2022). Bill 10 centralized and enhanced the decision-making powers of the province’s Health Minister and integrated regional agencies, which contained public health agencies, into 13 newly established territorial *Centres intégrés de santé et de services sociaux* (CISSS), which includes hospitals, primary healthcare organizations, public health, youth centres, and nine *Centres intégrés universitaires de santé et de services sociaux* (CIUSSS), which include university and health training centres (Wankah et al., 2018).

Ontario is a notable exception to this trend: efforts to centralize governance and decision-making of the health system have, to date, not included public health. Prior to 2019, key actors in Ontario’s health system were 34 local public health agencies (PHUs), responsible for public health programs and services, and 14 Local Health Integration Networks (LHINs), responsible for the organization and funding of healthcare services for geographically defined populations. In 2019, the Ontario Legislative Assembly passed *The People’s Health Care Act* to consolidate LHINs (except home and community care functions) and other healthcare agencies under a single provincial agency, Ontario Health, and to gradually establish Ontario Health Teams that would be accountable for providing a full continuum of patient-centred and community-based care services (Ontario Ministry of Health, 2019a). The Ontario Ministry of Health also proposed to “modernize” and improve coordination and integration between Ontario’s healthcare and public health systems, by consolidating public health agencies and bringing them under ten health regions, and reducing provincial contributions to public health agencies as part of the existing provincial-municipal funding arrangement (Financial Accountability Office (FAO) of Ontario, 2019; Ontario Ministry of Health, 2019b). While some public health agencies began to proactively adapt to the proposed financial and structural plans, broader structural public health system reform was paused due to the COVID-19 pandemic.

Given these varying experiences, the objective of this study was to understand how reforms to health system governance, organization, and financing have impacted public health systems in three Canadian provinces.

## Methods

### Study design

The study presented is an exploratory, comparative, multiple case study (Yin, 2014). Our approach to our study design, data collection, and analysis was guided by constructivist and pragmatic worldviews (Creswell, 2014; Kelly & Cordeiro, 2020). The key objective of our analysis was to understand the experiences, knowledge, and actions of those with experience with recent health system reforms and generate actionable knowledge useful for audiences within and beyond public health systems. This research was guided by the WHO’s essential enabling public health operations framework, which describes public health systems as the collection of entities and enabling structures that support public health operations (Rechel et al., 2018). An interview guide was piloted with members of a broader study working group made up of public health and health systems scholars and practitioners, and iteratively refined as needed throughout data collection.

### Data collection

We conducted semi-structured interviews between October 2020 and April 2021. To identify potential participants, study members reviewed the organizational charts of provincial and local public health organizations, membership lists of professional public health associations, and working group recommendations, and conducted manual internet searches. A combination of maximum variation and snowball purposive sampling was used to recruit participants (Palinkas et al., 2015). Participants were selected based on expertise and experience within their respective public health systems at the provincial and regional/local levels of governance. Participants were prompted to describe the public health systems in their jurisdiction, including which reforms they felt were the most significant in terms of impacts on public health in the province, and the impact that the reform had on the governance, organization, workforce, and/or financing of the public health system. Most interviews were attended by a secondary note-taker, and a debrief occurred after each interview to reflect on the interview and understandings of the participant’s experiences.

A total of 58 participants were interviewed (Alberta, *n* = 21; Ontario, *n* = 18; Québec, *n* = 19). A summary of participant characteristics from each province is presented in Table 1. Most participants were from public health agencies located in urban settings and represented a range of leadership positions within provincial and regional/local public health agencies. Interviews were approximately 60 min, conducted virtually through videoconferencing software, and audio recorded and transcribed verbatim. Interviews were conducted in French (for Québec) by a Francophone interviewer (MMS), and professionally translated and transcribed into English, then reviewed for accuracy. Interviews continued until the point of thematic saturation.

**Table 1:** Participant characteristics across three Canadian provinces (total, *N* = 58)

	Characteristic	Alberta (*n* = 21)	Ontario (*n* = 18)	Québec (*n* = 19)	Total (*N* = 58)
Geography (catchment area)	Urban	15	11	14	40 (69%)
	Rural/Northern	6	7	5	18 (31%)
Workplace	Local/Regional public health department or health authority	14	15	10	39 (67%)
	Provincial government or arm’s-length scientific institute	< 5	< 5	9	15 (26%)
	Other (e.g., federal health agency, professional associations, NGO)	< 5	< 5	< 5	4 (7%)
Discipline (registered profession or area of work)	Medical or nursing	11	10	10	31 (53%)
	Other (e.g., policy and management, dental, nutrition, health promotion, epidemiology, environmental, and occupational health)	10	8	9	27 (47%)
Role while employed in the public health system	Senior leadership (e.g., deputy minister, CMOH, CEO, provincial executive director)	6	5	5	16 (28%)
	Medical officer of health	6	5	6	17 (29%)
	Other (e.g., director, manager, program lead, consultant)	9	8	8	25 (43%)

### Data analysis

A preliminary coding guide was developed a priori by study team members with training and experience in qualitative methods (TJ, RWS, HSS), using the interview guide as a starting point. Directed content analysis was used primarily as the deductive approach for data analysis, and initial codes were refined as data analysis proceeded (Hsieh & Shannon, 2005). In the first phase of data analysis, three team members (TJ, RWS, HSS) independently coded two randomly selected transcripts from each province. Coding was reviewed for inter-coder agreement, and disagreements were resolved through discussion. Subsequent transcripts for each province were analyzed independently and were verified by a secondary coder. All data analysis was completed using NVivo (QSR International, version 12).

During the second phase of the study, a within and cross-case analysis was conducted to identify cross-cutting similarities and differences among the cases. Data were analyzed deductively and inductively using in-depth thematic analysis to iteratively conceptualize and refine themes (Braun & Clarke, 2006). TJ developed preliminary themes based on coded interview data and memos that reflected prominent perspectives within and across provinces. These preliminary themes were then independently examined by another study team member (RWS, HSS, MO), refined, and presented to the study team leads (SA, ADP) for review and further refinement. This process was repeated across all cases.

### Ethics

This study was approved by the University of Toronto Research Ethics Board (REB-39438). Informed consent was obtained from all individual participants included in the study. The Consolidated Criteria for Reporting Qualitative Research checklist (Online Resource 1) guided how we reported findings (Tong et al., 2007).

## Results

Our analysis yielded three themes that described the interrelated drivers and perceived impacts of health system centralization reforms, or proposed reforms, on public health systems and essential public health operations in Alberta, Québec, and Ontario. These themes include (1) Promising “value for money” and consolidating authority; (2) Impacting intersectoral and community-level collaboration; and (3) Deprioritizing public health operations and contributing to workforce precarity.

### Promising “value for money” and consolidating authority

Participants suggested that the rationale for reforms in each province centred around the potential health system cost-savings and efficiency improvements of consolidating decision-making authority and reorganizing health systems and public health operations that were perceived or framed as inefficient and administratively costly. Participants described political interests that propelled the rapid implementation of reforms without explicit intent to strengthen essential public health operations.

Several participants across provinces described fundamental differences between public health and healthcare sector operations not being well understood or valued by politicians and key decision-makers. This was most prominent in Québec, where participants reported that public health was framed as “*…more of an administrative service than clinical service*” (QC-08), and an area to save on health system costs:

“*[Minister Barrette] said ‘Let’s eliminate the regional level.’ except that the Public Health Director was in there so [Minister Barrette] was forced to recognize that they aren’t just bureaucrats who coordinate directives, they establish priorities, financial decisions and other, […] they carry out public health actions, but still they do a lot of administration, that’s where they cut [funding by] 30%. So, it was very misguided. There was no understanding of the public health function[s]*” (QC-01).

In Québec, participants described reforms as stemming from the provincial government’s intention to reduce the provincial deficit, Minister Barrette’s strong motivation to centralize decision-making authority under the Health Minister and Ministry of Health, and reduce funding pressure on the healthcare system:

“*A strong political will upheld by an extremely powerful political entrepreneur, Mr. Barrette, who wanted to centralize decision-making at [the provincial] level. […] This allowed them to have more authority on budget decisions and to have public health included in the government’s plan to reach deficit zero quickly, which they succeeded in, right?*” (QC-06).

“*... [the reform] was not even in the political platform of the party to which Mr. Barrette adhered. It was not discussed during an electoral campaign. It happened without there being any of the three proofs [scientific, administrative, or political], and why it happened, was because of the strong personality of the Minister who brought it in, because he was wise enough to push his reform at the very beginning of the mandate of a new government in the honeymoon period that accompanies a new government*” (QC-03).

A point of interest in Alberta was the government’s motivation to reduce inter-regional competition between powerful regional authorities, primarily those with jurisdiction over major urban centres:

“*[There was] less willingness to share the resources or to pool capacity or to develop things jointly between regions. So, I think there was a lot of frustrations at the political level […]so the Minister of the day and the Premier of the day made a decision to start from scratch, basically, and to start with a single health authority. And of course, you know, not everybody was happy*” (AB-09).

Other participants from Alberta also spoke about the motivations of cost-containment and streamlining decision-making authority, noting that “*the biggest overall thing, and the reason that they did it was for cost. They would have one […] CEO instead of one for each area, one for each zone, you know*” (AB-16). Participants stated that centralization helped to “streamline” leadership, reduce duplication, and standardize public health programs and services across regions by identifying where variation in service delivery existed. They also noted challenges that came with standardization:

“…*one of the first things that the new super health authority did was to ask, well, which programs are core? […] And anything that wasn’t delivered in every single part of the province got axed. And that included things that were in fact better than what was existing*” (AB-07).

In Ontario, some participants attributed interest in public health “modernization” as recognition from the government as an opportune time to better align Ontario’s public health sector with the healthcare system during a time of broader health system restructuring taking place within the province. One participant deliberated that “*I think they tried to tie organizational reform with funding reform. Like they thought, ‘perhaps if we are able to then merge these health units then we could save on financial costs at the same time.’ […] I think they were just applying that approach to [consolidate] local public health [agencies] without understanding the unique interface with local public health [agencies] and municipalities*” (ON-07). Participants noted that while consolidation of PHUs was framed as an opportunity to improve value for money, interest in doing so had been previously identified under the Liberal Party government as a strategy to address long-standing workforce capacity challenges and improve performance of public health functions (e.g., around chronic disease and injury prevention):

“*[…it] was an idea that was there. I think with the Conservative government, the idea aligned with trying to find some efficiencies and amalgamate governance bodies to try to decrease the amount spent on administrative staff or even back office that could be aligned*” (ON-01).

### Impacting intersectoral and community-level collaborations

Participants shared mixed perspectives on the impacts that health system centralization reforms had on the nature of engagement and capacity of public health actors to collaborate with health and non-health sectors, and relationships with local community-level partners.

In Alberta, participants reported that centralization helped to improve coordination and planning particularly between public health and healthcare sectors. One participant stated that:

“*…we’re able to kind of coordinate with the larger structure and […] have better alignment. And if we need to have resources moved around, we can do that. If there’s strategies that need to be co-developed, there’s more opportunity to do that*” (AB-04).

Participants also pointed to challenges in maintaining relationships between public health actors within the health authority and community-level actors (e.g., community-based organizations and municipalities), and responding to local community health needs, as:

“*…moving from those regional authorities to this centralized body in some ways like severed all those relationships. So, the regional health authorities would have had a really strong connection to the municipalities […] and municipal governments. […] But that doesn’t really happen here in the same way because everything that was in place to facilitate that kind of got broken and nothing replaced it in the way that you would hope to*” (AB-01).

Participants in Québec expressed unfavourable views about the impacts of the 2015 health system reform. In particular, some felt that the restructuring had not achieved a key objective of improving the continuum of patient care across health and social services. Sectors were described as continuing to operate in silos, and the reform resulted in the loss of regional resources which facilitated collaboration (e.g., program management positions) and in the transfer of some public health portfolios (e.g., prevention programs supporting mothers with substance use challenges and programs targeted towards infants and early years) to other departments beyond the responsibility of Public Health Directors:

“*…one would have expected that the structural integration in the CISSS/CIUSSS would have facilitated cooperation between the different missions of organizations, that is to say, cooperation between public health and the others… And we have seen that this was not really the case*” (QC-03).

Centralization was seen to sever connections and interaction between public health agencies and community-level actors with shared interests around population health and equity in Québec. One participant described “*…the atrophy of proximity. How we work with the local actors, and not just within health, but with the cities, community organizations…*” (QC-01), with public health departments experiencing greater challenges being, or becoming less, responsive to local needs. The former structure of Local Community Service Centres (les centres locaux de services communautaires) was seen to have:

“*[…] allowed a sharing of resources which contributed to tackling shared challenges, shared problems, which isn’t as possible now. It’s more difficult [to collaborate with local actors] now because we have to go through the local will now to achieve the collaboration, whereas before there was a dedicated resource that facilitated this collaboration*” (QC-16).

The proposed centralization reforms in Ontario raised concerns about losing local voices and partnerships, and municipal supports for public health operations. “*When it comes to the local concerns and issues, we have the relationships, we know who’s on the ground, we can make those connections immediately, we know who to connect out to, they know who to connect into at the local level*” (ON-11).

One participant reflected that the proposed consolidation of PHUs might mean that:

“…*the representation from our local councillors or citizens would be a lot less, would be a lot less local, because a governance body can only be so big. And the ability to have local representation on your governance board, governance body would be much more restricted*” (ON-09).

Several participants pointed out the proposed reforms would most significantly impact smaller and equity-seeking communities (e.g., rural communities, people experiencing income and housing insecurity) due to “*the loss of that closer relationship with the municipalities and the structure, [that might make it] more difficult to achieve such an important program or to initiate such an important program*” (ON-07).

### Deprioritizing public health operations and contributing to workforce precarity

Across jurisdictions, participants expressed concerns around decision-makers prioritizing healthcare services for investment over preventive public health services and programs. Participants described how centralization reforms involved funding reductions for public health requiring prioritization of specific public health operations, such as communicable disease control, and the continued whittling of the public health workforce.

In describing concerns regarding the proposed consolidation of local PHUs favouring investment in healthcare versus public health, one participant in Ontario stated that, “*[…] the larger issue that concerns the field about modernization […] is the idea that as soon as you combine public health with healthcare, because healthcare is a beast in terms of the resources that it currently uses, it will swallow up any smaller player […]*” (ON-18). In Alberta, “*[the Alberta Cancer Board] were spending at least $3 [to] 4 million dollars a year on tobacco control. But when that got pulled into Alberta Health Services, that money just disappeared. Like that went into the acute care structure*” (AB-06). Alternatively, a participant from a public health agency serving a rural/northern population in Québec voiced that integration within the CISSS structure conferred “*[…] more control on where [public health] money goes, a better uniformity of basic services in each of the localities*” (QC-07).

Participants in Québec and Ontario also described how funding changes and restructuring resulted in prioritizing specific public health operations. In Québec, participants saw health protection activities (e.g., communicable disease control) impacted less by the 30% budget cuts than health promotion. Some participants stated that because of the consolidation of the regional public health authorities into the local health system level, some programs were abandoned altogether due to budget reductions and responsibility for some core functions were ultimately not adopted at the provincial level as planned:

“*[The] goal was to consolidate things, but that’s not how it went in practice, they didn’t, for example, build a central team for the development of promotional materials…it’s like they took the resources, or cut the resources, but they didn’t take over the functions, that’s actually quite complex to do from a central position, so it wasn’t successful and slowly people went back to how they were doing it before, but never with the same capacity*” (QC-10).

Similarly, several Ontario respondents expected that funding changes accompanying announced reforms would result in prioritizing some program areas at the expense of others, particularly health promotion:

“*if [they] did get funding cut, [they would] have to cut staff. And unfortunately, it is very difficult to continue on with […] programs like chronic disease prevention or promotion where the impacts of [their] efforts are not seen for several years down the line*” (ON-07).

Furthermore, Ontario participants suggested that having more explicitly defined standards and more easily quantified objectives for practice in the areas of health protection versus health promotion (which includes activities addressing social determinants of health and health equity) may influence this prioritization:

“*[The] Ontario Public Health Standards [… are] extraordinarily explicit around the health protection function […] whereas the health promotion pieces are more about, well, depending on your local situation, work with your local partners and identify what needs to occur to improve the health of the population*” (ON-02)*.*

Across the three cases, participants described impacts of reforms on public health workforce and morale. For example, in Québec, impacts included the “*loss of knowledge, loss of expertise, loss of personnel, loss of jobs especially in management*” (QC-06). Participants also described the difficulty in replacing the shrinking public health workforce, further hindering the ability to continue some public health programs, which one participant described as “*a bit of a vicious cycle*” (QC-11). In Alberta, one participant stated that “*because of these budget cuts […] we’re losing capacity in public health*” (AB-06). In Ontario, the proposal to reduce the number of local public health agencies instilled insecurity within the public health workforce, causing the departure of qualified public health experts and positions in public health leadership to remain vacant prior to the COVID-19 pandemic. Participants also reported that the speed with which the new funding structure was announced and implemented did not allow for planning to mitigate potential workforce challenges*:*

“*… from a leadership perspective, [it is important] to keep our staff morale up and engaged. […] Who wants to come work in a local public health unit if you think, you know, that that’s going to be dissolved […] by the end of the year?*” (ON-09).

## Discussion

Prior to the COVID-19 pandemic, many provinces and territories in Canada had undergone major health systems reforms, moving towards centralized authority and consolidated delivery. These reforms had notable impacts on Canada’s public health systems. The drivers and impacts of health system reforms are, to various extents, related to each other. Our results illustrate how the promise of health system reforms were perilous for some areas of the public health system. Reforms were led by governments with conservative-leaning ideologies emphasizing cost-containment and reducing administrative inefficiency; they may ultimately have negatively impacted funding to public health, and they may have eroded core public health operations such as health promotion and the public health workforce. Furthermore, drivers for organizational reforms further centralizing authority may have impacted inter-sectoral collaboration and community-level partnerships.

Recent provincial health system reforms that consolidated authority to achieve stated goals of improved “efficiency” and “reduced administration” have been perceived by public health leaders to undermine some core public health operations and connections with local partners and communities. While governments face pressure to contain costs and return to pre-pandemic spending levels, overall health budgets continue to get squeezed and public health may continue to face more financial cuts over healthcare services (Di Ruggiero et al., 2022). The experiences of centralization reforms in Alberta and Québec suggest that core public health functions, such as health promotion, may be the most vulnerable to budget cuts. Our study suggests that these impacts may relate to the limited understanding of elected decision-makers with respect to the role and activities of the public health system, and/or a lack of willingness to prioritize preventive and population health equity approaches.

Like all public policy decisions, health system reforms are political decisions. If political will is needed to strengthen the role of public health and place system issues higher on political agendas, public health professionals might consider increasing political engagement and communicating to elected decision-makers the long-term economic benefits of investing in public health infrastructure and strategies (Greer et al., 2017). An often-cited barrier to these public health investments is the mismatch of timeframes between short political cycles for elected decision-makers under pressure to secure measurable and “quick wins,” and the longer timeframes required to demonstrate the health impacts and cost-effectiveness of public health interventions (Richardson, 2012). Richardson (2012), for example, offers potential solutions: first, encourage willingness among elected decision-makers and the public by emphasizing the short-term impacts, such as vaccination campaigns, and framing long-term gains of investing in public health as investments in future well-being. Second, Richardson (2012) suggests that using the criteria of “cost-effectiveness” rather than “cost-savings” would allow for some public health strategies to be evaluated using the same criteria used to evaluate treatment interventions. Without the recognition of the key distinctions between healthcare and public health, and the need to ensure strong connections with local populations, concerns about weakened public health systems will continue to persist (Khaleghian & Gupta, 2005). As decision-makers continue to seek ways to improve efficiency and remove silos within the health system, inclusive decision-making and collaborative governance could help ensure appropriate expertise and contribution to resource allocation, policy, and planning processes.

Comparing the experiences of reforms across three provinces also allows us to draw some lessons for policymakers interested in following similar paths towards more centralized health systems, such as the Government of Newfoundland and Labrador (Government of Newfoundland & Labrador, 2022). Our findings support some of the theorized benefits of centralizing authority, such as some improved coordination of activities between public health and healthcare, and standardization of practices, as reported in Alberta. However, these benefits must be weighed against the unintended harms of centralization reforms, such as a reduction in accountability as well as in proximity and responsiveness to local-level need (Abimbola et al., 2019). The challenges faced in Alberta and Québec with respect to maintaining effective intersectoral action both locally and provincially suggest that these concerns should be explicitly addressed when restructuring health systems. Furthermore, the pre-pandemic proposal to consolidate local public health agencies in Ontario raises critical questions about how local public health agencies might mitigate the potential loss of local and community-level partnerships. At the same time, such consolidation holds some promise among public health leaders for improved coordination of public health programs, and the potential to address long-standing challenges experienced by smaller and/or rural public health agencies, such as filling vacancies in senior leadership positions (e.g., Medical Officers of Health), securing funding, and providing infrastructure supports.

The COVID-19 pandemic has sparked renewed interest in supporting and strengthening provincial and territorial public health systems in Canada. Recent research examining how centralization and integration reforms may have facilitated and impeded COVID-19 response (Smith et al., 2023), and early impacts of the pandemic on public health systems in these provinces (Sandhu et al., 2022), provide some insights and future considerations for public health. Moreover, our findings also reflect the stated priorities of the Chief Public Health Officer of Canada in the 2021 annual report, “*Vision to Transform Canada’s Public Health System*” to: ensure public health’s role in governance discussions, strengthen intersectoral connections, increase public health funding that matches the mandate of public health and remains consistent, and recruit and retain a skilled workforce (Chief Public Health Officer of Canada, 2021). It is clear that, 20 years later, many of the recommendations proposed in the “*Learning from SARS: Renewal of public health in Canada*” report, such as federal funding to support and strengthen core public health functions in provinces and territories, developing a national public health strategy that defines and monitors core activities, and developing a strategy for renewing human resources in public health, have yet to be adopted (Government of Canada, 2003).

This work highlights some of the drivers and impacts of broader health system reforms on public health systems and services from the perspectives of those leading and working in public health. It also provides preliminary insights for further research into the inter-relationships between the drivers and the impacts of these reforms.

### Limitations

We were able to interview a diverse sample of public health leaders in three distinct public health systems; however, we are missing a broader range of perspectives from front-line public health workers, as well as other health system stakeholders. As many participants were in key public health leadership roles at the time of the interviews, responses may have been affected due to their position within public health. To help mitigate this, we collected a range of insights from people no longer working within the public health system, and leaders at different levels of seniority. Therefore, future work could include the perspectives of other public health professionals and representatives of key actors who partner with or use public health programs and services who may provide a more holistic assessment of centralization reforms.

While recruiting participants, we sought diverse representation of identities and experiences; however, recording sociodemographic information could have helped assure this goal and deepen the relevance and trustworthiness of our findings. Our study does not comprehensively reflect the diverse experiences of public health leaders, particularly those working within First Nations, Inuit, and Métis communities and organizations. Furthermore, our study was not designed from the outset as per First Nations Ownership, Control, Access, and Possession (The First Nations Information Governance Centre, 2014) or Inuit Tapiriit Kanatami (Inuit Tapiriit Kanatami, 2018) principles for ethical research. To mitigate potential harm from this oversight, interview data were offered back to one participant who self-identified as an Indigenous community member.

Finally, our study was conducted during an acute phase of the COVID-19 pandemic, which may have impacted the perceptions of public health leaders and their ability to reflect on the pre-pandemic health systems reform. Ongoing public health systems research and attention to public health perspectives will help gain additional insights to support decision-makers in their efforts to strengthen health systems and ultimately improve population health.

## Conclusion

Major reforms to health systems broadly aiming to centralize, consolidate, or standardize health system operations and functions have taken place recently across Canada, but how they have impacted the public health system remained unclear. This study provides insights into how centralization reforms impacted public health system governance, organization, financing, and practice. Specifically, from the perspectives of public health leaders, health system reforms that broadly aimed to consolidate authority, contain costs, and improve efficiency have (1) shifted resources towards healthcare sectors; (2) presented challenges to intersectoral and local-level collaboration; and (3) eroded some public health operations and the public health workforce. Our findings highlight opportunities for reform strategies to leverage the strengths of centralization while minimizing limitations of centralization. While our work does not represent the full range of experiences of reforms within public health systems in Canada, by considering the lessons learned from the provinces of Alberta, Ontario, and Québec, we hope that our unifying themes present important considerations for decision-makers and health system leaders interested in implementing similar reforms.

## Contributions to knowledge

What does this study add to existing knowledge?

- This is the first comparative case study that has examined the impact of major centralization reforms on public health systems and essential public health operations from the context of Canadian public health leaders. This work adds to the limited literature on the impacts of reforms on public health systems, which may help to inform other jurisdictions considering similar centralization reforms.
- As limited evidence currently exists as to whether large-scale structural reforms have been successful in the Canadian context, these findings also present an opportunity to evaluate whether centralization reforms have achieved their intended goals.

What are the key implications for public health interventions, practice, or policy?

- These findings present areas for consideration about the impacts of centralization reforms on public health operations and the public health workforce, and the need to consider and mitigate unintended consequences of health systems reform on public health.
- This study highlights the need for stable investment in public health systems to support public health functions, and for increased advocacy from public health leaders.
- Further deliberation around centralization should consider from where, provincially or locally, core programs are delivered.

## Supplementary Information

Below is the link to the electronic supplementary material.

Supplementary file1 (DOCX 32 KB)

## Data Availability

Available upon request.

## Code availability

None to declare.

## References

[CR1] Abimbola S, Baatiema L, Bigdeli M (2019). The impacts of decentralization on health system equity, efficiency and resilience: A realist synthesis of the evidence. Health Policy and Planning.

[CR2] Allin, S., Sherar, M., Peckham, A., & Marchildon, G. (2018). *Province-wide services* (Rapid Review No. 10). North American Observatory on Health Systems and Policies. Retrieved October 9, 2021, from https://naohealthobservatory.ca/research/rapid-review-10/

[CR3] Arpin, E., Smith, R. W., Cheung, A., Thomas, M., Luu, K., Li, J., Allin, S., Rosella, L., Pinto, A. D., & Quesnel-Vallée, A. (2022). *Profiles of public health systems in Canada: Québec*. National Collaborating Centre for Healthy Public Policy. Retrieved June 14, 2022, from https://ccnpps-ncchpp.ca/docs/2022-Profiles-of-Public-Health-Systems-in-Canada-Quebec.pdf

[CR4] Braun V, Clarke V (2006). Using thematic analysis in psychology. Qualitative Research in Psychology.

[CR5] Breton M, Lévesque J-F, Pineault R, Lamothe L, Denis J-L (2009). Integrating public health into local healthcare governance in Quebec: Challenges in combining population and organization perspectives. Healthcare Policy.

[CR6] Chief Public Health Officer of Canada. (2021). *The Chief Public Health Officer of Canada’s report on the state of public health in Canada 2021: Vision to transform Canada’s public health system*. Public Health Agency of Canada. Retrieved January 18, 2022, from https://www.canada.ca/en/public-health/corporate/publications/chief-public-health-officer-reports-state-public-health-canada/state-public-health-canada-2021.html

[CR7] Creswell, J. W. (2014). Qualitative methods. In *Research design: Qualitative, quantitative and mixed methods approaches* (4th ed., pp. 183–213). SAGE Publications Inc. 10.5539/elt.v12n5p40

[CR8] Di Ruggiero, E., Bhatia, D., Umar, I., Arpin, E., Champagne, C., Clavier, C., Denis, J.-L., & Hunter, D. (2022). *Governing for the public’s health: Governance options for a strengthened and renewed public health system in Canada*. National Collaborating Centres for Public Health. Retrieved June 14, 2022, from https://nccph.ca/projects/canadas-chief-public-health-officer-2021-report-and-associated-commissioned-reports/governing-for-the-publics-health-governance-options-for-a-strengthened-and-renewed-public-health-system-in-canada/

[CR9] Duckett S (2010). Second wave reform in Alberta. Healthcare Management Forum.

[CR10] Fierlbeck, K. (2019). Amalgamating provincial health authorities: Assessing the experience of Nova Scotia. *Health Reform Observer – Observatoire Des Réformes de Santé*, *7*(3), Article 3. 10.13162/hro-ors.v7i3.4046

[CR11] Financial Accountability Office (FAO) of Ontario. (2019). *Expenditure estimates 2019–20: Ministry of Health and Long-Term Care*. Financial Accountability Office (FAO) of Ontario. Retrieved July 8, 2022, from https://www.fao-on.org/web/default/files/publications/1902%20Estimates%20MOHLTC/FA1902%20Estimates%20Review%202019-20%20MOHLTC.pdf

[CR12] Government of Canada & Naylor, C. D. (Eds.). (2003). *Learning from SARS: Renewal of public health in Canada: A report of the National Advisory Committee on SARS and Public Health*. National Advisory Committee on SARS and Public Health. Retrieved February 10, 2023, from https://www.canada.ca/content/dam/phac-aspc/migration/phac-aspc/publicat/sars-sras/pdf/sars-e.pdf

[CR13] Government of Newfoundland and Labrador. (2022). *Transitional CEO announced for single health authority planning phase*. News Releases. Retrieved July 8, 2022, from https://www.gov.nl.ca/releases/2022/health/0425n01/

[CR14] Greer SL, Bekker M, De Leeuw E, Wismar M, Helderman JK, Ribeiro S, Stuckler D (2017). Policy, politics and public health. European Journal of Public Health.

[CR15] Hoffman SJ, Creatore MI, Klassen A, Lay AM, Fafard P (2019). Building the political case for investing in public health and public health research. Canadian Journal of Public Health.

[CR16] Hsieh H-F, Shannon SE (2005). Three approaches to qualitative content analysis. Qualitative Health Research.

[CR17] Inuit Tapiriit Kanatami. (2018). *National Inuit strategy on research*. Retrieved July 8, 2022, from https://www.itk.ca/projects/national-inuit-strategy-on-research/

[CR18] Kelly LM, Cordeiro M (2020). Three principles of pragmatism for research on organizational processes. Methodological Innovations.

[CR19] Khaleghian P, Gupta MD (2005). Public management and the essential public health functions. World Development.

[CR20] List of Alberta general elections. (2022). In *Wikipedia*. Retrieved February 10, 2023, from https://en.wikipedia.org/w/index.php?title=List_of_Alberta_general_elections&oldid=1129071088

[CR21] List of Ontario general elections. (2022). In *Wikipedia*. Retrieved February 10, 2023, from https://en.wikipedia.org/w/index.php?title=List_of_Ontario_general_elections&oldid=1125422189

[CR22] List of Québec general elections. (2022). In *Wikipedia*. Retrieved February 10, 2023, from https://en.wikipedia.org/w/index.php?title=List_of_Quebec_general_elections&oldid=1116051300

[CR23] Ontario Ministry of Health. (2019a). Ontario Health Teams: Guidance for health care providers and organizations. Retrieved February 9, 2023, from https://www.publications.gov.on.ca/CL29930

[CR24] Ontario Ministry of Health. (2019b). *Discussion paper: Public health modernization*. Queen’s Printer for Ontario. Retrieved October 9, 2021, from https://www.health.gov.on.ca/en/pro/programs/phehs_consultations/docs/dp_public_health_modernization.pdf

[CR25] Palinkas LA, Horwitz SM, Green CA, Wisdom JP, Duan N, Hoagwood K (2015). Purposeful sampling for qualitative data collection and analysis in mixed method implementation research. Administration and Policy in Mental Health.

[CR26] Quesnel-Vallée, A., & Carter, R. (2018). Improving accessibility to services and increasing efficiency through merger and centralization in Québec. *Health Reform Observer – Observatoire Des Réformes de Santé*, *6*(1), Article 1. 10.13162/hro-ors.v6i1.3216

[CR27] Rechel, B., Jakubowski, E., McKee, M., & Nolte, E. (2018). *Organization and financing of public health services in Europe*. WHO Regional Office Europe on behalf of the European Observatory on Health Systems and Policies. Retrieved September 26, 2021, from https://www.euro.who.int/en/publications/abstracts/organization-and-financing-of-public-health-services-in-europe-2018 30620513

[CR28] Richardson AK (2012). Investing in public health: Barriers and possible solutions. Journal of Public Health.

[CR29] Sandhu HS, Smith RW, Jarvis T, O’Neill M, Di Ruggiero E, Schwartz R, Rosella LC, Allin S, Pinto AD (2022). Early impacts of the COVID-19 pandemic on public health systems and practice in 3 Canadian provinces from the perspective of public health leaders: A qualitative study. Journal of Public Health Management and Practice.

[CR30] Smith RW, Jarvis T, Sandhu HS, Pinto AD, O’Neill M, Di Ruggiero E, Pawa J, Rosella L, Allin S (2023). Centralization and integration of public health systems: Perspectives of public health leaders on factors facilitating and impeding COVID-19 responses in three Canadian provinces. Health Policy.

[CR31] Smith, R. W., Allin, S., Rosella, L., Luu, K., Thomas, M., Li, J., & Pinto, A. D. (2021). *Profiles of public health systems in Canada: Ontario*. National Collaborating Centre for Healthy Public Policy. Retrieved June 14, 2022, from https://ccnpps-ncchpp.ca/profiles-of-public-health-systems-in-canadian-provinces-and-territories/

[CR32] Smith, R. W., Allin, S., Luu, K., Li, J., Jarvis, T., Thomas, M., Li, J., Rodrigues, A., Rosella, L., & Pinto, A. D. (2022). *Profiles of public health systems in Canada: Alberta*. National Collaborating Centre for Healthy Public Policy. Retrieved June 14, 2022, from https://ccnpps-ncchpp.ca/profiles-of-public-health-systems-in-canadian-provinces-and-territories/

[CR33] The First Nations Information Governance Centre. (2014). *Ownership, control, access and possession (OCAP)*. The First Nations Information Governance Centre. Retrieved July 8, 2022, from https://www.deslibris.ca/ID/10095457

[CR34] Tong A, Sainsbury P, Craig J (2007). Consolidated criteria for reporting qualitative research (COREQ): A 32-item checklist for interviews and focus groups. International Journal for Quality in Health Care.

[CR35] Wankah P, Guillette M, Dumas S, Couturier Y, Gagnon D, Belzile L, Mosbah Y, Breton M (2018). Reorganising health and social care in Québec: A journey towards integrating care through mergers. London Journal of Primary Care.

[CR36] Yin RK (2014). Case study research: Design and methods.

