# Supplementary material for: Promise and peril: how health system reforms impacted public health in three Canadian provinces
Source: Can J Public Health. 2023 Jul 6;114(5):714–25. doi: 10.17269/s41997-023-00785-2 (PMC10484823; doi:10.17269/s41997-023-00785-2)
Supplement: Supplementary file 1 — Supplementary file1 (DOCX 32 KB) [file 41997_2023_785_MOESM1_ESM.docx]

**Article title**: Promise and peril: How health system reforms impacted public health in three Canadian provinces

**Journal name**: Canadian Journal of Public Health

**Author names**: Tamika Jarvis^1,2^, Robert W. Smith^3^, Harman Singh Sandhu^4^, Muriel Mac-Seing^5^, Meghan O’Neill^6^, Laura Rosella^6,7,8,9^, Sara Allin^2,4^, Andrew D. Pinto^10,11,12,13*^

**Affiliations**:

1. Department of Health Research Methods, Evidence, and Impact, Faculty of Health Sciences, McMaster University, Hamilton ON, L8S 4L8
2. North American Observatory on Health Systems and Policies, Dalla Lana School of Public Health, University of Toronto
3. Division of Social and Behavioural Health Sciences, Dalla Lana School of Public Health, University of Toronto
4. Institute of Health Policy, Management and Evaluation, Dalla Lana School of Public Health, University of Toronto
5. Centre for Global Health, Dalla Lana School of Public Health, University of Toronto
6. Population Health Analytics Lab, University of Toronto, Toronto, Ontario, Canada
7. Laboratory Medicine and Pathobiology, Temerty Faculty of Medicine, University of Toronto
8. Institute for Clinical Evaluative Sciences
9. Institute for Better Health, Trillium Health Partners
10. Upstream Lab, MAP/Centre for Urban Health Solutions, Li Ka Shing Knowledge Institute, Unity Health Toronto
11. Department of Family and Community Medicine, Faculty of Medicine, University of Toronto
12. Department of Family and Community Medicine, St. Michael’s Hospital
13. Division of Clinical Public Health, Dalla Lana School of Public Health, University of Toronto

**Corresponding author***: Andrew D. Pinto, MD, MSc, Upstream Lab, MAP Centre for Urban Health Solutions, Li Ka Shing Knowledge Institute, Unity Health Toronto. 30 Bond Street, Toronto, Ontario, Canada, M5B 1W8. 416-864-6060 x76148 ([andrew.pinto@utoronto.ca](mailto:andrew.pinto@utoronto.ca)).

**Online Resource 1:** Modified consolidated criteria for reporting qualitative research (COREQ) checklist and notes.

| **No.** | **Item** | **Guide Questions/Description** | **Notes** | **Page Number (if applicable)** |
| --- | --- | --- | --- | --- |
| **Domain 1: Research team and reflexivity** | | | | |
| **Personal Characteristics** | | | | |
| 1. | Interviewer/facilitator | Which authors conducted the interview or focus group? | The interviews were conducted by authors TJ, RWS, SS and research team members MMS and MS. | N/A |
| 2. | Credentials | What were the researcher's credentials? *E.g. PhD, MD* | Authors have a range of credentials: MPH, MD, PhD | N/A |
| 3. | Occupation | What was their occupation at the time of the study? | The researchers held a variety of roles as graduate students, researchers, university faculty members, professors, and public health practitioners. | Page 2 |
| 4. | Gender | Was the researcher male or female? | We believe the gender of the researchers is not relevant to discuss in detail for this study. | N/A |
| 5. | Experience and training | What experience or training did the researcher have? | There was a range of experience and training present. A few of the authors were graduate students who had taken courses in qualitative methods and analysis and there were also researchers who had multiple years of combined theoretical training and application in case study and qualitative methods. | N/A |
| **Relationship with participants** | | | | |
| 6. | Relationship established | Was a relationship established prior to study commencement? | In a small number of cases, study team members had an existing professional relationship with the participants which facilitated recruitment. | N/A |
| 7. | Participant knowledge of the interviewer | What did the participants know about the researcher? e*.g. personal goals, reasons for doing the research* | Participants were provided information about the study objectives and interview questions ahead of their interview as part of receiving informed consent. | Page 4 |
| 8. | Interviewer characteristics | What characteristics were reported about the interviewer/facilitator? e.g. *Bias, assumptions, reasons and interests in the research topic* | None reported. | N/A |
| **Domain 2: study design** | | | | |
| **Theoretical framework** | | | | |
| 9. | Methodological orientation and Theory | What methodological orientation was stated to underpin the study? *e.g. grounded theory, discourse analysis, ethnography, phenomenology, content analysis* | This qualitative study was underpinned by constructivist and pragmatic principles. | Page 2 |
| **Participant selection** | | | | |
| 10. | Sampling | How were participants selected? *e.g. purposive, convenience, consecutive, snowball* | This is described in the methods section. Participants were selected through a combination of purposive and snowball sampling approaches. | Page 2 |
| 11. | Method of approach | How were participants approached? e*.g. face-to-face, telephone, mail, email* | The participants were approached through email. | N/A |
| 12. | Sample size | How many participants were in the study? | This is described in the methods section (total *n* = 58). | Page 4 |
| 13. | Non-participation | How many people refused to participate or dropped out? Reasons? | No participants dropped out. | N/A |
| **Setting** | | | | |
| 14. | Setting of data collection | Where was the data collected? e*.g. home, clinic, workplace* | The data was collected virtually through Zoom and/or Microsoft Teams software. | Page 4 |
| 15. | Presence of non-participants | Was anyone else present besides the participants and researchers? | In one case, there was the presence of an executive assistant along with the participant. The rest of the interviews only included the participants and researchers. | N/A |
| 16. | Description of sample | What are the important characteristics of the sample? *e.g. demographic data, date* | These are described in Table 1 of the manuscript. | Page 18 |
| **Data collection** | | | | |
| 17. | Interview guide | Were questions, prompts, guides provided by the authors? Was it pilot tested? | The interview guide was pilot tested internally with the research team and advisory working group members. The interview questions can be requested through the corresponding author. | N/A |
| 18. | Repeat interviews | Were repeat interviews carried out? If yes, how many? | No repeat interviews were carried out. | N/A |
| 19. | Audio/visual recording | Did the research use audio or visual recording to collect the data? | Audio recording was collected through Zoom and/or Microsoft Teams software. | Page 4 |
| 20. | Field notes | Were field notes made during and/or after the interview or focus group? | Notes and memos were made following the interview to capture reflections and an audit trail has been maintained. | Page 4 |
| 21. | Duration | What was the duration of the interviews or focus group? | The interviews lasted for 60 minutes on average. | Page 4 |
| 22. | Data saturation | Was data saturation discussed? | Data saturation was only considered at the point of data collection. We assumed, *a priori*, that conducted 15-20 interviews for each province would provide us with a saturation of perspectives and themes. | Page 4 |
| 23. | Transcripts returned | Were transcripts returned to participants for comment and/or correction? | The transcripts were not returned to the participants. | N/A |
| **Domain 3: analysis and findings** | | | | |
| **Data analysis** | | | | |
| 24. | Number of data coders | How many data coders coded the data? | There were three total data coders (authors TJ, RWS, HSS). | Page 4 |
| 25. | Description of the coding tree | Did authors provide a description of the coding tree? | A brief description of the coding approach (through directed content analysis) is provided. | Page 4 |
| 26. | Derivation of themes | Were themes identified in advance or derived from the data? | Themes were derived from the data. | Page 4 |
| 27. | Software | What software, if applicable, was used to manage the data? | NVivo was used to manage and analyze the data. | Page 4 |
| 28. | Participant checking | Did participants provide feedback on the findings? | Synthesized member checking was conducted. | Page 11 |
| **Reporting** | | | | |
| 29. | Quotations presented | Were participant quotations presented to illustrate the themes/findings? Was each quotation identified? e*.g. participant number* | Quotations and participant numbers are provided in the results section. | N/A |
| 30. | Data and findings consistent | Was there consistency between the data presented and the findings? | The themes presented are consistent with the quotations used. | N/A |
| 31. | Clarity of major themes | Were major themes clearly presented in the findings? | All themes presented were major since they were cross-cutting and salient in the data. A balanced description of the themes is provided. | N/A |
| 32. | Clarity of minor themes | Is there a description of diverse cases or discussion of minor themes? | N/A | N/A |
